# Bioinformatics Tools for Small Genomes, Such as Hepatitis B Virus

**DOI:** 10.3390/v7020781

**Published:** 2015-02-16

**Authors:** Trevor G. Bell, Anna Kramvis

**Affiliations:** Hepatitis Virus Diversity Research Programme (HVDRP), Department of Internal Medicine, School of Clinical Medicine, Faculty of Health Sciences, University of the Witwatersrand, Johannesburg 2050, South Africa; E-Mail: Anna.Kramvis@wits.ac.za

**Keywords:** sequence data, sequence fragments, chromatograms, DNA assembly, amplicons, indels, alignment, phylogenetics, GenBank

## Abstract

DNA sequence analysis is undertaken in many biological research laboratories. The workflow consists of several steps involving the bioinformatic processing of biological data. We have developed a suite of web-based online bioinformatic tools to assist with processing, analysis and curation of DNA sequence data. Most of these tools are genome-agnostic, with two tools specifically designed for hepatitis B virus sequence data. Tools in the suite are able to process sequence data from Sanger sequencing, ultra-deep amplicon resequencing (pyrosequencing) and chromatograph (trace files), as appropriate. The tools are available online at no cost and are aimed at researchers without specialist technical computer knowledge. The tools can be accessed at http://hvdr.bioinf.wits.ac.za/SmallGenomeTools, and the source code is available online at https://github.com/DrTrevorBell/SmallGenomeTools.

## 1. Introduction

The analysis of DNA sequence data is a routine procedure in many laboratories. The workflow from sample to final sequence alignment is often similar in many studies and consists of several steps involving bioinformatic manipulation of biological data. The software available for bioinformatic analysis ranges from complex, integrated analysis suites, which run on all major operating system platforms, to highly-specialized command-line tools, which require compilation from source-code on selected operating systems only, to online services available on the Internet, which run in a web browser on any software platform. Available programs and online resources may be generic and able to process sequence data from any organism or may be specific to a particular subset of organisms. Resources may be aimed at general users or at technical researchers with specialist skills. The technical aspects of biological data analysis may be challenging for biologists, who may not possess specialist, technical computer skills. We present here a suite of online bioinformatics tools aimed at the non-technical user. Most are genome-agnostic and can be used with sequence data from any organism, whilst two are specific to hepatitis B virus (HBV), which has a small, circular DNA genome, of approximately 3,200 nucleotides. The tools described here supplement tools to explore sequence variation and residue distribution [1], to merge long overlapping sequence fragments [2] and to process and interrogate ultra-deep pyrosequencing data [3].

## 2. Implementation and Discussion

### 2.1. Overview

The tools presented in this paper are available online on the Internet, at no cost, at http://hvdr.bioinf. wits.ac.za/SmallGenomeTools. Technical notes are available via a link at the same address. The tools themselves make use of a module written in the Python programming language (http://python.org), which has been described previously [1,2]. The source code for the tools is released under the GPL version 2 and is available online via GitHub, at https://github.com/DrTrevorBell/SmallGenomeTools.

A standard laboratory workflow in our laboratory [4] includes DNA extraction, PCR amplification, direct DNA sequencing by a third party service, viewing and checking of chromatograms, preparation of curated sequences, multiple sequence alignment, sequence analysis, serotyping, genotyping, phylogenetic analysis and preparation of sequences for submission to GenBank. During these processes, challenges were encountered in the analysis of HBV sequence data, and bioinformatic tools were developed to address these (Table 1). Stand-alone, web-based tools allow users on any operating system platform to access the tools they require from any location with an Internet connection, without needing to learn a new bioinformatics software suite or a new program and without having to install any software onto their computer. The appropriate tool is simply used as and when required.

**Table 1:** Online tools developed and the workflow process for which each would be used.

Workflow	Tool Name	Description	Source	Input	Performance
Chromatograms	Quality Score Analyzer	Plots Chromatogram Quality Scores	Sanger	Chromatogram	0.4 s for 1200 bases
Chromatograms	Automatic ContigGenerator Tool (ACGT)	Generates a contig from a forward and reverse chromatogram	Sanger	Chromatogram	0.5 s for two chromatograms of 300 bases each
Alignment	Automatic Alignment Clean-up Tool (AACT)	Eliminates “gap-columns” and disambiguates ambiguous bases	Sanger NGS *	FASTA	0.2 s for 3800 sequences of 3221 bases in length (12-MB file)
Alignment	Mind the Gap	Splits FASTA file based on gap threshold per column	Sanger NGS	FASTA	0.5 s for 3800 sequences of 3221 bases in length (12-MB file)
Analysis	Babylon Translator	Extracts HBV protein sequences (ORFs)	Sanger NGS	FASTA	0.9 s for 3800 sequences of 3221 bases in length (12-MB file)
Analysis	Wild-type 2 × 2	Calculates 2 × 2 wild-type/mutant contingency tables	Sanger NGS	FASTA	0.1 s for two groups of 50 sequences each of 3221 bases in length
Serotyping	HBV Serotype Tool	Determines HBV Serotype	Sanger NGS	FASTA FASTA	0.6 s for 225 sequences of 3221 bases in length
Phylogenetics	Pipeline: TreeMail	Generates a phylogenetic tree	Sanger	Phylip	1000 bootstraps of 41 sequences of 1000 bases required 15 min to process and email
GenBank Preparation	PadSeq Tool	Places two HBV sequence fragments on a template	Sanger	FASTA	0.6 s to place 3800 sequences from each of two input files

* Performance given in seconds (s), excluding network-dependent file uploading and page downloading times; NGS is next generation sequencing, such as ultra-deep pyrosequencing.

### 2.2. Quality Score Analyzer

**Figure 1:**
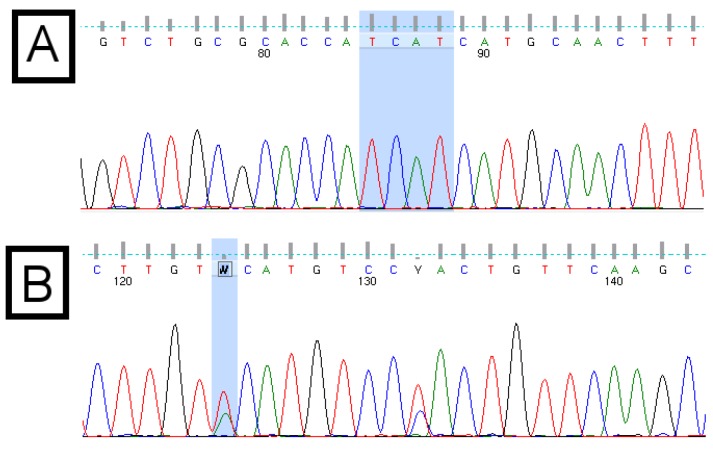
Example chromatograms of the basic core promotor/precore (BCP/PC) region of the hepatitis B virus (HBV) genome. Panel (**A**) shows the “Kozak” region (TCAT) of subgenotype A1, followed by the “ATG” pre-core start codon; Panel (**B**) shows ambiguous (wobble) bases, which result from double peaks. These indicate a mixed population or the presence of quasispecies. Quality scores are indicated by the grey bars above each base call. The quality score associated with the ambiguous “W” base in panel (**B**) is 15, compared with scores above 50 for each base of the “TCAT” motif in panel (**A**).

Direct DNA sequencing of PCR amplicons is a routine laboratory procedure. The result of this sequencing reaction is a chromatogram file, which is also known as a trace file or an electrophoretogram (visualized in Figure 1). A common file format for chromatograms is the Applied Biosystems format (ABIF; The ABIF file format specifications are available online at http://www.appliedbiosystems.com/support/software_community/ABIF_File_Format.pdf), which has a file name extension of “ab1”. In a process known as “base calling”, which is typically part of the DNA sequencing service, each nucleotide in the sequence is automatically identified by a software program. A “quality score”, implemented originally by the “phred” base-calling program, is assigned to each base call [5,6]. This score, which is logarithmic, indicates the reliability or confidence of the base call. A value of 10 indicates a one-in-10 (90%) probability that the base call is incorrect. A value of 20 indicates a one-in-100 (99%) probability of an incorrect base call. Generally, quality scores greater or equal to 20 are considered reliable. Due to the nature of sequencing reactions, quality scores at the beginning and the end of chromatograms are generally too low to be considered reliable and are therefore routinely removed before any downstream processing is done.

The quality scores of the base calls in a chromatogram are important. In some cases, the overall quality of an entire chromatogram is so poor, that it should not be used. In other cases, regions of the chromatogram are of poor quality. Using a poor quality chromatogram in an application or online tool will typically result in poor quality results or no results. An online tool was developed to assist users to determine the overall quality of a chromatogram file.

**Figure 2:**
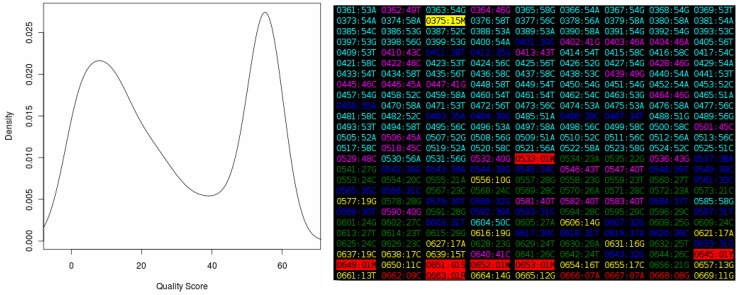
The output of the “Quality Score Analyzer” tool, showing the density plot on the left and a section of the “heat map” on the right. Each entry in this map is in the format “XXXX:YYZ”, where “XXXX” is the base position number in the sequence, increasing from “0001” for the first position in the file, and “YY” is the quality score from the chromatogram. The “Z” is the base called at the position. The color of each entry represents the quality score. Values in the range zero to nine (considered very poor) are shown in red, between 10 and 19 (poor) in yellow, between 20 and 29 (acceptable) in green, between 30 and 39 (good) in blue, between 40 and 49 (very good) in magenta and between 50 and 59 (excellent) in cyan. Quality scores higher or equal to 60, which are theoretical only, are shown in white. Ambiguous bases are shown in reverse colors (black text on a colored background).

The online “Quality Score Analyzer” requires an “ab1” chromatogram file as input and displays a box-and-whisker plot (*not shown*) and density plot of the quality scores and a “heat map” (Figure 2). The “heat map” provides a visual representation of the overall quality of an entire chromatogram. Areas of interest, such as regions of low quality, can be examined in more detail. No trimming of the input chromatogram is performed.

### 2.3. Automatic Contig Generator Tool

To ensure accurate coverage of a DNA region, a PCR amplicon may be sequenced in both the forward (5’ to 3’) and the reverse direction. A consensus sequence of the forward and reverse reads can be produced by a number of proprietary software programs or by checking both chromatograms and editing the sequence data manually, which is time consuming and error prone.

The “Automatic Contig Generator Tool” (ACGT) generates a consensus sequence (“contig”) from a forward and reverse chromatogram file. Each chromatogram is trimmed (In this implementation, the front-end uses default trimming parameters, which the user cannot adjust. The “load” method of the “Sequence” class provides adjustable trimming parameters. Details of these, and the method used to trim chromatograms, are discussed elsewhere [1].), and the sequence designed as “reverse” is reversed and complemented. A pairwise alignment, using the BioPython [7] wrapper for the “needle” alignment algorithm [8] is then executed. The tool then constructs a consensus sequence by examining the pairs of bases at each position in turn and determining which base to include. The tool generates a consensus from a forward and reverse sequence of the same amplicon region; it is not used to assemble overlapping regions. The quality of each residue and the average quality over a user-defined window of bases are used to determine which residue should be retained. The default value for the window is to include five bases downstream and five bases upstream of the current residue. The tool determines which of the two residues to include by applying the following rules:
**Algorithm 1:** Algorithm for the Automatic Contig Generator Tool (ACGT).
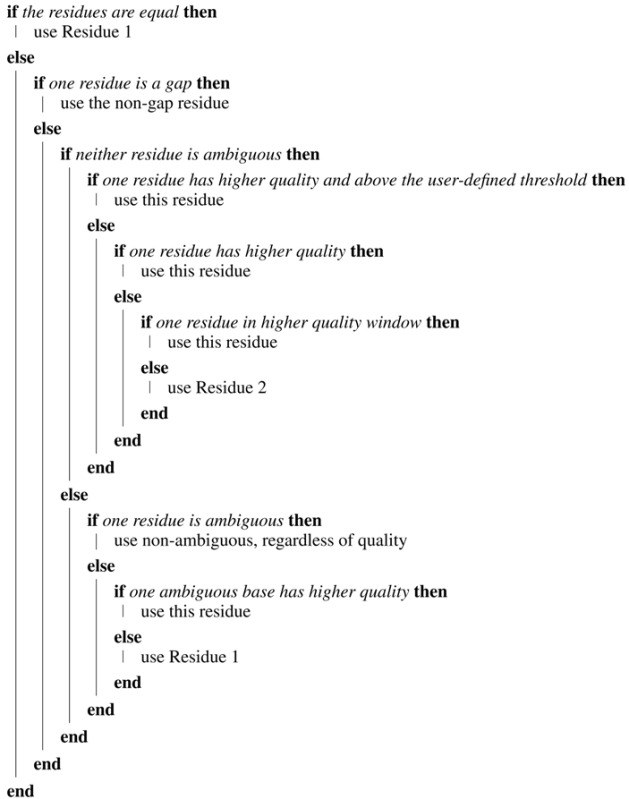


**Figure 3:**
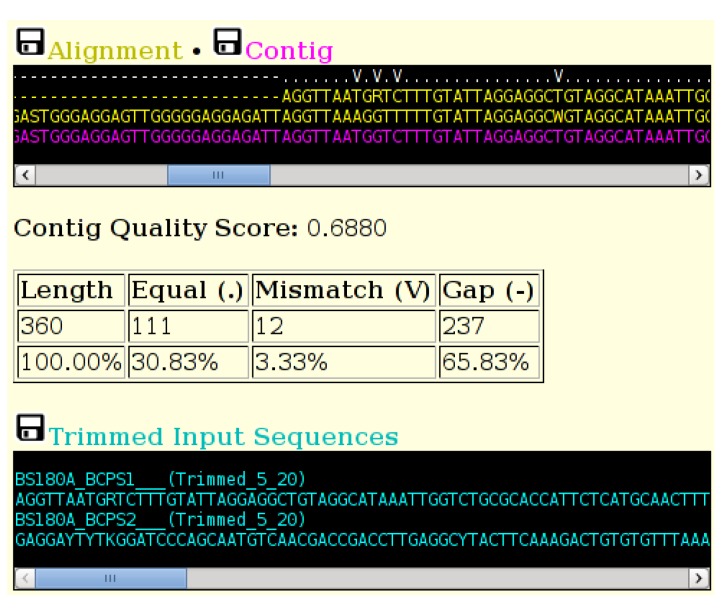
A section of the output of the ACGT tool, showing the indicator line (top) in white, the two trimmed and aligned sequences (yellow) and the computed consensus sequence (magenta). The table displays totals of the various consensus groups. The trimmed sequences are displayed in cyan. Sequences can be downloaded in FASTA format by selecting the small black disk icon.

The output of the tool is shown in Figure 3. A “consensus level” indicator line is included above the alignment. This line shows different characters depending on the level of consensus: both bases equal, bases mismatched or one base is a gap. The characters used can be specified on the main input screen. The font size of the sequence output can also be specified. A consensus “quality score” is calculated by adding to a running total a value of one when both residues are equal or a value of 0.5 if one position is a gap. The final total is divided by the length of the consensus to give the consensus quality score. A table below the contig box summarizes the various consensus levels in the alignment. The trimmed input sequences are included below the table for reference. The alignment, contig and trimmed sequences can be downloaded in FASTA format.

### 2.4. Automatic Alignment Cleanup Tool

When working with sequence data from several samples, it is routine to prepare a multiple sequence alignment. The data in this alignment will often require careful checking and curating before being used in further analyses. Whilst automated curation is difficult and may incorrectly manipulate the sequence, there are instances where some automatic correction may be possible. The “Automatic Alignment Clean-Up Tool” (AACT) performs one or both of the following two actions on sequences in an aligned FASTA file.

(1) Eliminate “gap-columns”: Columns (positions) in the multiple sequence alignment, which contain at least the specified threshold (expressed as a percentage) of gaps, will be eliminated from the sequence. This is intended to remove “misreads”, which occur often, but not exclusively, in homopolymeric regions and result in only one or two bases in a column, which consists only of gaps otherwise.
(2) Disambiguate gap-free “mono-base” columns: Ambiguous bases in a column, which consists otherwise of only one base type and is free of gaps, are disambiguated from the base in the column if the ambiguous base represents the base in the column. For example, if a column contains base “A”, except for one sequence, which contains an “M” (“A” or “C”), this “M” will be disambiguated from “A”. If the sequence contained a “Y” (“C” or “T”) instead of an “M”, this position would not be disambiguated. All positions are converted to uppercase characters.

The user can specify which of the two components (or both) should be executed on the input file. A report is displayed showing the details of how the alignment was changed. A link is provided to download the “cleaned” FASTA file.

**Figure 4:**
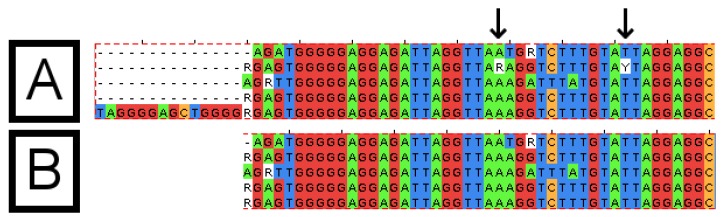
Sequence alignments before and after processing by the Automatic Alignment Clean-Up Tool (AACT). The sequence alignment, which was provided as input to the "AACT" tool (**A**) and the alignment produced by the tool (**B**). Columns containing at least 80% gaps (top, left of image) have been removed. Columns containing only one base type, with an ambiguous base, have been disambiguated (arrows). Columns containing a variety of bases are not altered. The alignments were visualized using the “JalView” program.

Additionally, the user may specify that the start and/or end of the sequences be “blunted”, such that all columns containing running gaps from the start of the sequence and/or to the end of the sequence are removed. Every sequence in the alignment, therefore, starts and/or ends with a base, rather than a gap. If both ends are blunted, the alignment is trimmed to the length of the shortest sequence. Alignments, using the “JalView” program (http://www.jalview.org/), before and after processing by the tool, are shown in Figure 4. The tool also outputs various numerical results, including the number of gap-columns, which were eliminated.

It is recommended that this tool only be used after the alignment has been examined carefully, as the tool may potentially remove mutations from the sequence. However, only ambiguous bases or residues in “gap-columns” would be removed in this way. The suggested approach would be to use the tool to clean an alignment instead of doing such cleaning manually. Submitting uncurated and unchecked data to the tool may result in deletions or changes to the sequence, which are undesirable or incorrect.

### 2.5. Mind the Gap

Multiple sequence alignments may contain columns, which consist mainly of gaps. This typically occurs when one or a few sequences in the alignment contain an additional base (insertion), particularly in homopolymeric regions of ultra-deep pyrosequencing data [3]. The “Mind the Gap” tool splits an input FASTA file into two files, based on the percentage of gaps present at one or more columns in the alignment. If the number of gaps in a column exceeds the threshold (percentage) specified, all sequences with the gap will be placed into one FASTA file (the gaps will be removed first), and all sequences containing the residue (the insertion) will be placed into a second FASTA file. This is repeated for all remaining sequences, with sequences being added to the files, so that only two output files are produced, with and without insertions, respectively. Both FASTA files are provided for download on the output page of the tool.

### 2.6. Babylon Translator

**Figure 5:**
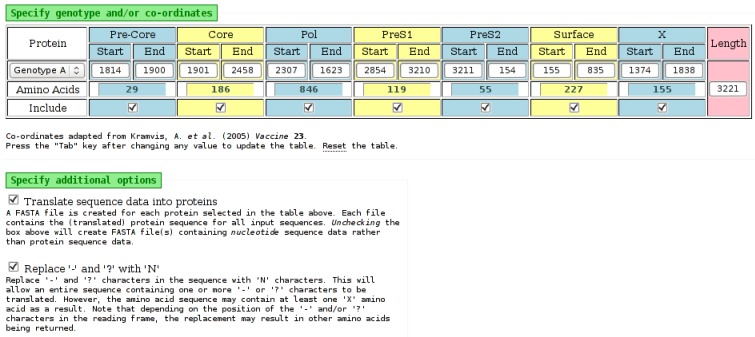
Part of the input page of the “Babylon” tool. Selecting a genotype from the list on the left will populate the nucleotide positions for each protein with default values from [10]. However, each of these positions can be edited, as necessary. The number of amino acids for each protein is determined automatically from the nucleotide values. An “Include” field for each protein specifies if it should be included in the output. The amino acid output is obtained by selecting the appropriate check-box on the input page. The “-” and “?” characters, which may be present in input sequence data, will be processed by the tool as an “N” character if the appropriate check-box is selected. It may be possible to translate nucleotides to amino acids when “-” and “?” characters are replaced with “N” characters.

The HBV genome codes for seven proteins, in four overlapping open reading frames (ORF) [9]. The “Babylon” tool extracts (splits) HBV sequence data, from a single input file, into multiple files, with each output file containing either nucleotide or translated amino acid data for one HBV protein, as specified on the input page. The tool does not require full-length sequences, and if required, the co-ordinates used to extract the protein/s can be specified manually. The tool processes a FASTA file, which should contain aligned nucleotide sequence data from samples belonging to a single HBV (sub)genotype. The input page of the “Babylon” tool is shown in Figure 5.

The tool extracts sequence data for each of the selected proteins from the FASTA file, optionally translating the data into amino acids, if specified. A separate output file (in FASTA format) is created for each selected protein, containing the nucleotide or amino acid data for all samples, for that protein only. The files can be downloaded individually or all together in one compressed archive (“ZIP”) file.

### 2.7. Wild-Type 2 × 2

When analyzing a set of HBV sequences, it is often desirable to compare the number of wild-type residues at a locus with the number of mutant (non-wild-type) residues at the same locus. In this case, “wild-type” refers to the residue, which occurs in the majority of the sequences. The “Wild-type 2 × 2” tool requires a FASTA file of (aligned) nucleotide or amino acid data as input. It calculates wild-type/mutant 2 × 2 contingency tables for sequences in the two specified groups, for all loci. Detailed output for loci, which are statistically significant at the specified threshold, is provided.

The input sequence data must be allocated into two groups using the number (numerical position) of sequences in the FASTA file. For example, if a file contains 20 sequences, with the first five representing “Group 1” (for example, sequences from males) and the remaining 15 representing “Group 2” (for example, sequences from females), this would be specified as “1–5” and “6–20”, without the quotation marks. Groups may also be specified as individual numbers, such as “1,3,6,7,10”, or as a mixture of both notations, such as “2,5,6–12”. No spaces or other characters are permitted. If one of the groups is omitted entirely (left blank), all sequences that are not allocated to the other group will automatically be allocated.

**Figure 6:**
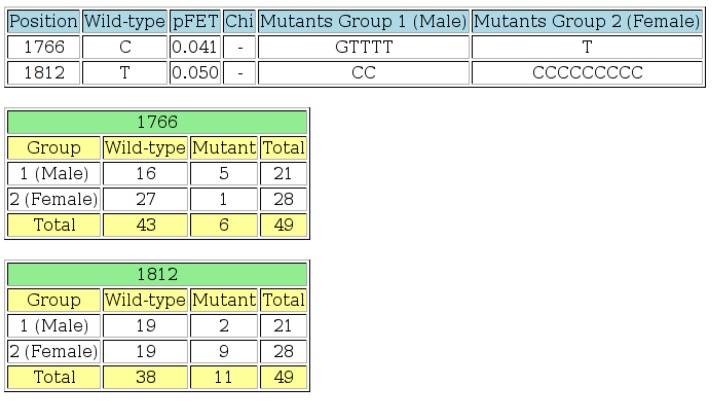
The output page of the “Wild-type 2 × 2” tool. The first table shows positions at which the *p*-value is less than or equal to the threshold specified, the wild-type residue, the Fisher’s exact test *p*-value (“pFET”), a list of the mutant residues found in Group 1 and a list of the mutant residues found in Group 2. At least one value in the 2 × 2 contingency table for each row in the table was less than or equal to five, so chi-squared tests were not performed in these cases. An offset value was specified on the input page, so the positions on the output page are adjusted accordingly. The next section of output shows the 2 × 2 contingency tables for each position listed in the first table. This output indicates that there is a statistically-significant difference at the 5% level, between HBV isolates from males and females at positions 1766 and 1812 of the basic core promotor (BCP) region.

For each position/locus in the sequence data, the majority residue (nucleotide or amino acid) is determined, and this is considered the “wild-type” residue for that locus. The number of mutant residues, at each position, is then determined. A 2 × 2 contingency table is constructed, for each position, using wild-type and mutant counts, for each of the two groups. If at least one cell in the table contains a value less than or equal to five, a Fisher’s exact test is performed; otherwise a chi-squared test is performed on the table data. If the resulting *p*-value is less than or equal to the threshold value specified on the input page, that position is considered as statistically significant, and the details of that position are included on the output page. The value of the optional “offset”, as entered on the input page, is added to the position in the output. This can be used to obtain output positions, which correspond exactly with genome co-ordinates. The two groups can be allocated names by entering text into the appropriate box on the input page. Example output is shown in Figure 6.

### 2.8. HBV Serotyper Tool

In addition to genotypic classification, HBV samples can be classified into one of nine serological subtypes (serotypes) [10,11]. This classification is determined by the amino acids present at either three or five known positions within the HBV surface antigen (HBsAg) [12,13,14,15]. The HBV serotype is loosely correlated with genotype [16]. A published decision tree (tabulated in Table 2) summarizes the interpretation of the amino acid positions to determine the HBV serotype [15].

**Table 2:** The decision tree, from [15], represented as a table. For each serotype, the required amino acids are indicated in the appropriate column. The numbered columns indicate the amino acid position within HBsAg.

Serotype	122	160	127	159	140
adr	K	R			
adw2	K	K	P		
adw3	K	K	T		
adw4	K	K	I or L		
ayr	R	R			
ayw3	R	K	T		
ayw4	R	K	I or L		
ayw1	R	K	P	A	
ayw2	R	K	P	Not A	Not S
ayw4	R	K	P	Not A	S

**Figure 7:**
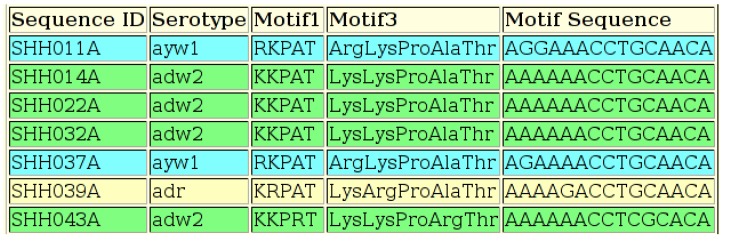
Output of the HBV Serotyping Tool, showing the sequence ID, the serotype, the amino acid motif for all five positions using both the one-letter amino acid abbreviations (“Motif1”) and the three-letter abbreviations (“Motif3”) and the nucleotides present at all five amino acid positions. All five amino acids are not required in order to deduce some serotypes, but all five positions are included for all samples for reference.

The “HBV Serotyper Tool”, based on this decision tree, requires a FASTA file of nucleotide data as input. This file should contain one or more aligned sequences, which must include the start of the HBsAg sequence and the amino acid positions required for serotyping. The input page of the tool presents a default nucleotide motif for the start of the S gene, which may be edited by the user, if necessary. The serotype, amino acid motif and nucleotide sequence are output (Figure 7).

### 2.9. Pipeline: TreeMail

Phylogenetic analyses undertaken by members of our research group typically involve several programs from the “Phylip” suite [17]. These command-line tools are interactive and menu-driven, requiring the user to undertake several steps to complete an analyses. An output data file from one component of the suite must be renamed manually to prepare it for use as an input file for another component of the suite. This process is repetitive and time consuming, especially when running several analyses.

**Figure 8:**
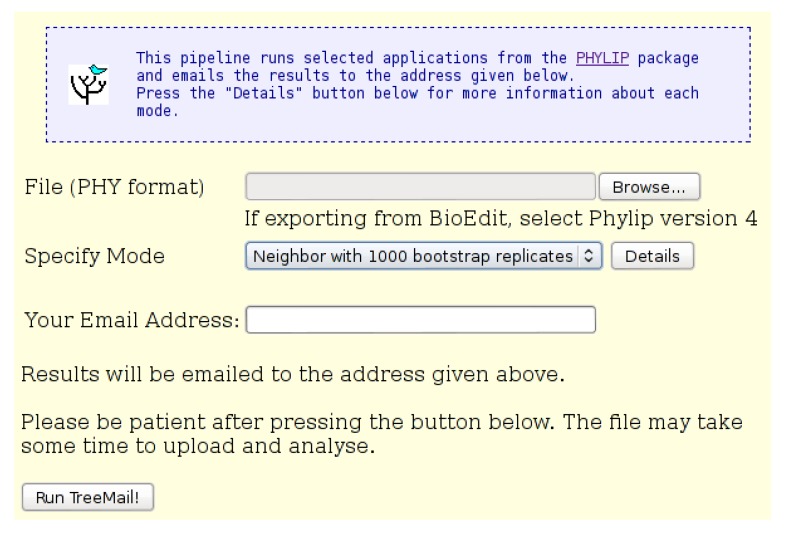
The “Pipeline: TreeMail” input page. An input file in “Phylip” format is required. The user may select either “Neighbor” mode, which does not include bootstrap replicates, or “Bootstrap” mode, which includes 1000 bootstrap replicates. Results of the analysis, including the tree file, are emailed to the address provided.

The “Pipeline: TreeMail” tool runs the “Phylip” *dnadist* and *neighbor*programs on the input file, with parameters automatically set as required by the research group (see below). The input page of the tool is shown in Figure 8. The input file must be in Phylip (“.phy”) format. The Kimura and lower-triangular settings are specified for *dnadist*, and the lower-triangular setting is specified for *neighbor*. The pipeline tool emails the resulting tree file (“.tre”) to the email address provided. The tool reports progress once the input file has been uploaded. A description of each of the “Phylip” programs used by the “Pipeline: TreeMail” tool is provided in Table 3.

If the “Bootstrap” mode is specified on the input page, the tool runs the Phylip *seqboot* program before *dnadist* and *consense* after *neighbor*, as described in the online documentation for *seqboot* http://evolution.genetics.washington.edu/phylip/doc/seqboot.html. When in “Bootstrap” mode, the pipeline tool will create 1000 datasets with *seqboot* and will email the final consensus tree to the email address provided.

The tool also emails the final Phylip “outfile” from either *neighbor* (normal mode) or *consense* (bootstrap mode) as a second attachment, called “result.txt”. When running in bootstrap mode, the actual bootstrap values will appear on the tree in this file (“result.txt”) and are present in the consensus tree file. These are shown as values out of 1000, not percentages.

**Table 3:** Programs from the “Phylip” suite, which are used by the “Pipeline: TreeMail” tool.

Program	Description
*consense*	Computes consensus trees using the majority-rule method
*dnadist*	Computes distances between samples from sequence data
*neighbor*	Computes an unrooted by neighbor-joining or UPGMA
*seqboot*	Generates multiple datasets by bootstrap resampling

Running in bootstrap mode may increase the time required for the tool to complete. A bootstrap test run of 41 sequences of approximately 1000 nucleotides each took 15 min to process and email. The web-page will time-out if the analysis takes longer than 60 min.

### 2.10. PadSeq Tool

Subgenomic fragments, particularly those less than 200 nucleotides in length, may not be accepted by GenBank. Such fragments can be submitted, in FASTA format, to the “PadSeq Tool”, which places each of two sequence fragments at the specified co-ordinates on a sequence backbone (template/scaffold) of the specified length. The tool can be used to generate artificial “full-length” sequences from two fragments, such as BCP/PC and S region fragments. This may be useful when attempting to genotype samples automatically.

**Figure 9:**
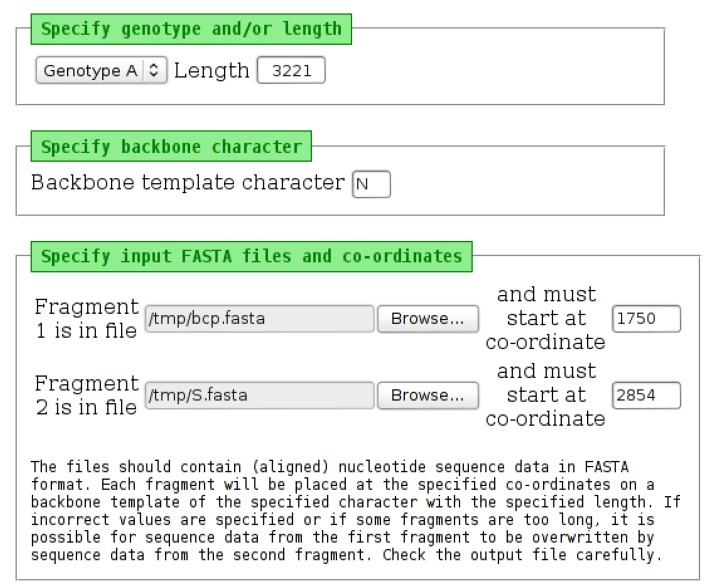
The input page of the “PadSeq Tool”. Selecting a genotype from the list will place a default length into the “Length” field. This value can be edited, if necessary. The backbone character can be changed from the default “N”. Each of the two input files must be specified, along with the starting position (co-ordinate) at which each fragment should be placed. The tool will “wrap” sequence data, which extends beyond the length specified, as may be required when processing HBV sequence data.

**Figure 10:**
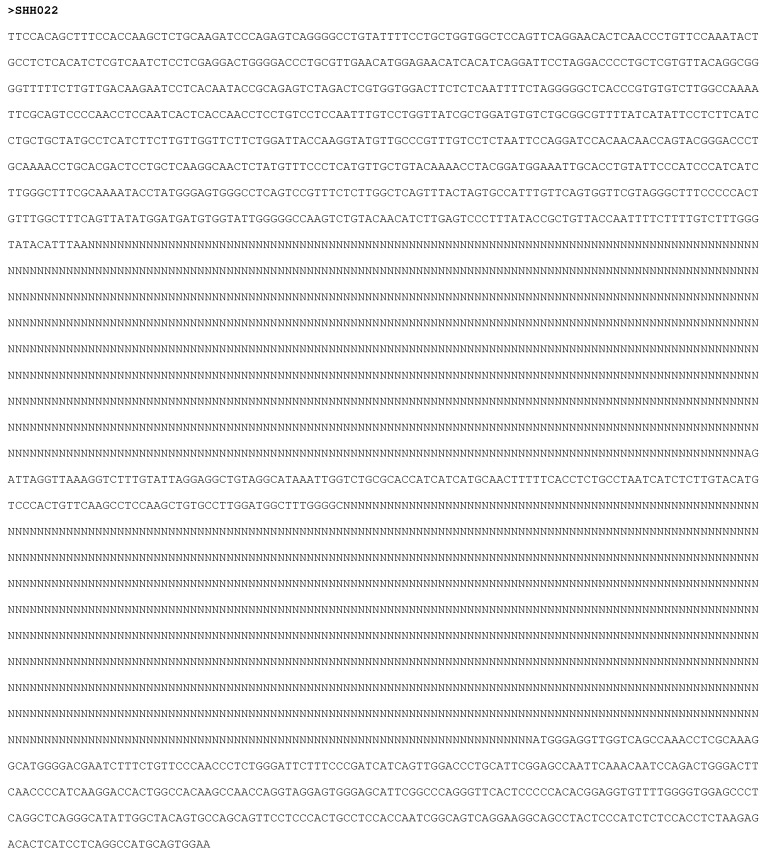
Example output of the “PadSeq” tool. The two input fragments have been placed at the specified location within a backbone template.

The tool requires two input files (Figure 9). One file should contain all of the sequence data for the first fragment, with each sequence in the file starting at the same co-ordinate. For example, this would be a file containing curated/checked basic core promotor/pre-core (BCP/PC) sequences, all starting at position 1750 from the *Eco*RI site. The second file should contain data for the second fragment, also all starting at the same position for the second fragment. For example, this would be a file containing curated/checked S region sequences all starting at position 2854 from the *Eco*RI site. The sequences do not all have to be the same length, but all of the sequences in one file must start at the same position. The order of the sequence data in both files must be the same. For example, if the BCP/PC file contains sequence data for Sample 1, then Sample 2, then Sample 3, the S region file must contain sequence data in the same order. The number of sequences in both files must be the same. Once the tool has completed, an output page is displayed, which contains a link to download one “padded” file of all sequence data in FASTA format. Example output is shown in Figure 10.

## 3. Conclusions

The suite of online tools presented here were developed in response to the direct needs of laboratory researchers working with DNA sequence data. They are available online at no cost and do not require extensive computer skills or training to use. Data can easily be processed by a mixture of online tools and other software packages, as standard file formats are used. Using specific tools, designed to perform a single task, means that workflows can be partitioned into logical units and that processes or analyses can be easily repeated.
